# Cholesterol of lipid rafts is a key determinant for entry and post-entry control of porcine rotavirus infection

**DOI:** 10.1186/s12917-018-1366-7

**Published:** 2018-02-12

**Authors:** Xiujing Dou, Yang Li, Junlan Han, Dante S. Zarlenga, Weijuan Zhu, Xiaofeng Ren, Na Dong, Xunliang Li, Guangxing Li

**Affiliations:** 1 Northeast Agricultural University, No. 59 Mucai Street, Xiangfang District, Harbin, 150030 China; 2 Animal Parasitic Diseases Laboratory, Agricultural Research Service, United States Department of Agriculture, Building 1180, BARC-East, Beltsville, MD 20705 USA

**Keywords:** Porcine rotavirus (PRV), Lipid rafts, Cholesterol, Methyl-β-cyclodextrin (MβCD), Infection, Entry

## Abstract

**Background:**

Lipid rafts are major structural components in plasma membranes that play critical roles in many biological processes including virus infection. However, few reports have described the relationship between lipid rafts and porcine rotavirus (PRV) infection. In this study, we investigated whether or not the locally high concentrations (3–5 fold) of cholesterol present in lipid rafts are required for PRV infection, and further examined which stages of the infection process are most affected.

**Results:**

When cellular cholesterol was depleted by methyl-β-cyclodextrin (MβCD), PRV infectivity significantly declined in a dose-dependent manner. This inhibition was partially reversed upon reintroduction of cholesterol into the system. This was corroborated by the co-localization of PRV with a recombinant, GPI-anchored green fluorescent protein, which functioned as a marker for membranous regions high in cholesterol and indicative of lipid rafts. Changes in virus titer and western blot analyses indicated that depletion of cellular cholesterol with MβCD had no apparent effect on PRV adsorption; however, depletion of cholesterol significantly restricted entry and post-entry of PRV into the cell. Both points of inhibition were restored to near normal levels by the addition of exogenous cholesterol.

**Conclusions:**

We conclude from these studies that membrane-based cholesterol and in particular that localized to lipid rafts, is an indispensable biomolecule for PRV infection, and that cholesterol-based control of the infection process takes place during entry and immediately post-entry into the cell.

## Background

Porcine rotavirus (PRV) is the most important cause of severe diarrhea in newborn piglets, and results in significant economic loss to pig industry. To date, efficient drugs for preventing PRV infection have not been identified. PRV, which belongs to the genus *Rotavirus* in the family *Reoviridae*, is a non-enveloped virus formed by three concentric layers of proteins [1]. The outermost layer consists of spike-like structures comprised of an unglycosylated VP4 protein [2] and a glycoprotein VP7, which forms the smooth surface of the virus. VP7 is a calcium-binding protein that interacts with cell receptors after the initial attachment of the virus to the cell surface [3].

It remains unclear as to how non-enveloped viruses which lack an outer lipid membrane infect host cells. It is generally believed that membrane fusion is not involved in virus entry and that release of these viruses is mainly performed by cell lysis [4]. It has been proposed that non-enveloped viruses penetrate the membrane barrier, either by the formation of a pore at the cell surface, or via the endocytic pathway using clathrin-coated vesicles or caveolae [4]. Alternatively, studies with simian virus 40 (SV40) [5] and poliovirus [6] have suggested that non-enveloped viruses are transported to specific domains within the plasma membrane using a vesicular transport mechanism, followed by replication in large inclusions in the cell cytoplasm or viroplasm [7]. Other biochemical and structural studies have shown that the overall mechanism is similar to enveloped viruses, and that viral capsid proteins may function in a manner analogous to the membrane viral proteins [4].

In recent years, research has exposed a crucial role for lipid rafts in a variety of virus life cycles [6–8]. Lipid rafts are traditionally described as microdomains within plasma membranes that are high in cholesterol, sphingolipids, glycosyl-phosphatidylinositol (GPI)-anchored proteins, and a specific set of associated proteins often cell receptors. They are thought to provide organization to and stabilize the structure of cell membranes because the high concentration of sphingolipids and protein receptors are densely packed together with cholesterol [9]. Although they are more ordered and closely packed, they still have the ability to float freely within the membrane bilayer. The most commonly used drugs to control the formation and function of lipid drafts are nystatin and filipin which sequester cholesterol, MβCD which is used to deplete membrane-bound cholesterol, and lovastatin which inhibits cholesterol biosynthesis [8].

Recent reports have demonstrated the involvement of lipids in a variety of cellular functions such as apical cell-sorting of proteins, signal transduction, caveolae mediated endocytosis, and viral release, assembly and budding in the cell membrane. These have been well documented in human immunodeficiency virus (HIV) [9], measles virus [10], influenza virus [11–13] and rotavirus [7, 14]. Lipids have also been deemed important in intracellular trafficking of viral proteins [15–18] (e.g. echovirus types 1 and 11, Ebola [19]), and provide platforms for cell entry (e.g. SV40 [20], HIV [21–24], echovirus type 1 [25] and rhesus rotavirus (RRV) [14]). Although it has been reported that RRV depends upon lipid rafts for cell entry [14], the specific role of cholesterol in these processes of PRV remains elusive.

Herein, we explored the importance of cellular cholesterol during PRV infection of MA104 cells by demonstrating that viral protein and genomic RNA levels were reduced significantly following MβCD treatment to deplete cellular cholesterol. We further showed that viral protein and genomic RNA levels were restored by subsequently treating the cells with exogenous cholesterol. Data showing the co-localization of VP7 with recombinant, fluorescently-labeled GPI-anchored protein linked the virus predominantly to lipid rafts. In as much as cholesterol was indispensable for both PRV entry and the post-entry process, lipid rafts could be a target for development of anti-PRV strategies.

## Methods

### Cells, viruses, antisera and reagents

MA104 and BHK-21 cells (obtained from China Center For Type Culture Collection) were cultured in Dulbecco’s Modified Eagle Medium (DMEM) (Gibco) supplemented with 10% fetal calf serum (FCS) at 37 °C under 5% CO_2_. PRV (strain DN30209, isolated in a pig farm, in Heilongjiang province of China) [26] and vesicular stomatitis virus (VSV, strain Indiana, obtained from prof. Joerg Glende, University of Veterinary Medicine Hannover, Germany) were propagated on the MA104 cells and BHK-21 cells, respectively, as previously described [26, 27]. In as much as previous reports showed that VSV infection on BHK-21 is not affected by depletion of cholesterol [27], we used VSV as a negative control for our studies about relationship between lipid rafts and virus infection.

Rabbit polyclonal antibodies to VP4 and VP7 were generated in our laboratory using previously described methods [26, 28]. Anti-VSV G-protein polyclonal antibody was purchased from Abcam. An anti-β-actin monoclonal antibody was purchased from Beyotime. The secondary antibodies were purchased from BD Biosciences. Both MβCD and cholesterol were purchased from Sigma and reconstituted in DMEM and alcohol, respectively.

### Virus titration

Approximately 1 × 10^4^ MA104 or BHK-21 cells were seeded onto 96-wells plates then incubated with purified PRV or VSV (100 μL virus/well) that was serially-diluted 10-fold prior to infection. Forty-eight hours after infection, the 50% tissue culture infectious dose (TCID_50_) of each well was determined according to the method of Reed and Muench by observing cytopathic effects (CPE). All experiments were conducted 5 times.

### Cytotoxicity assay

Toxic effects of MβCD and cholesterol on cells were determined using the Mosmann based assay (MTT) [29]. Cells were plated onto 96-well plates and upon reaching 80% confluency, they were incubated with different concentrations of MβCD (0, 2, 4, 6, 8, 10, 15 mM) or cholesterol (0, 50, 100, 150, 200 μM) for 48 h. Mock-treated cells served as controls.

### Depletion and replenishment of cholesterol from cells

For cholesterol depletion, cell monolayers were grown to 80% confluency, washed three times with cold phosphate-buffered saline (PBS), and then treated with various concentrations of MβCD ranging from 2 to 15 mM in serum-free DMEM for 30 min at 37 °C. Control cells were similarly treated but received no MβCD. All cells were subsequently washed with cold PBS. For cholesterol replenishment, washed cells were treated for 1 h at 37 °C with DMEM-soluble exogenous cholesterol at final concentrations ranging from 50 to 200 μM. Again the cells were washed extensively with PBS then subjected to virus (100 TCID_50_) infection.

### Indirect immunofluorescence assay (IFA)

At 24 h post-infection, all cells were washed and fixed with cold 1% paraformaldehyde for 30 min, and then balanced out by glycine 5 min. The cells were washed and permeabilized with 1% Triton X-100 for 10 min, and then incubated with 200 μL polyclonal antibody against the PRV VP7 protein or VSV-G protein at 37 °C for 45 min. After washing, the cells were incubated with an optimum dilution of fluorescein (FITC)-conjugated goat anti-rabbit IgG (H + L) (OriGene, Beijing) for 30 min at 37 °C then washed again in the dark, and examined under a fluorescence microscope (Zeiss Axiovert 200).

### Co-localization of PRV VP7 and GPI-anchored protein

MA104 cells were seeded onto 24-well tissue culture plates (2 × 10^5^ per well) and transfected using lipofectamine 2000 (Invitrogen, USA) with the pEGFP-GPI, a DNA plasmid encoding GPI-tethered to the enhanced green fluorescent protein (provided by Kai Simons, Max-Planck-Institute of Molecular Cell Biology and Genetics, Germany). At 6 h post-transfection, cells were infected with PRV (100 TCID_50_) for 24 h at 37 °C. Polyclonal antibody to PRV VP7 protein was used for virus detection by IFA using a fluorescence microscope.

### Real-time PCR

Real time-PCR was used to evaluate and quantify the level of viral infection following treatment. Briefly, total genomic RNA was extracted from viruses using RNA extract reagent (Fastgene, China) and cDNA was synthesized using M-MLV reverse transcriptase (Takara Bio Inc., Shiga, Japan). The cDNA was PCR amplified and the products were quantified using SYBR green as fluorescence dye (Takara Bio Inc., Shiga, Japan). The data were analyzed with ABI PRISM 7500 SDS software. Gene quantification was performed using the 2^-ΔΔCT^ method. The primer sequences are shown in Table 1.

**Table 1:** The primer sequences used for real-time PCR

Gene	Primer sequence (5′ to 3′)	
VP7	Forward	TGTCCTCTAAATACGCAGACTC
	Reverse	CTACCTGAATTACAGCGACAT
β-actin	Forward	GGCTCAGAGCAAGAGAGGTATCC
	Reverse	GGTCTCAAACATGATCTGAGTCATCT
VSV-G	Forward	TTCAAGCAGACGGTTGG
	Reverse	TGCTTCGGCATCCGTCA

### Western blot

The cells were harvested and lysed with cold cell lysis buffer containing 1% Triton X-100 and 1% protease inhibitor (Beyotime Institute of Biotechnology, USA). Total lysate was separated on a 12% SDS-PAGE gel then transferred to nitrocellulose membranes. The membranes were blocked with 5% non-fat dry milk in Tris-buffered saline (10 mM Tris-Cl at pH 7.5 and 150 mM NaCl) containing 0.05% Tween 20 (TBST) at 37 °C for 1 h, and then incubated with primary antibody at room temperature for 4 h. Detection was performed using horseradish peroxidase (HRP)-linked secondary antibody at room temperature for 1 h. Blots were developed using enhanced chemiluminescence detection system (Applygen, China).

### Stage specificity of cholesterol effects on PRV infection

To investigate the effects of cholesterol depletion on PRV infectivity in MA104 cells, the cell monolayers were incubated with 8 mM or 15 mM MβCD for 30 min at 37 °C, then washed. Virus (100 TCID_50_) was inoculated onto the cells at 4 °C where only adsorption can occur. After 2 h, the unbound virus was removed by washing and the medium replaced with DMEM. The cells were cultured until control cells exhibited complete CPE. For cholesterol replenishment, cells were pretreated with 15 mM MβCD at 37 °C for 30 min, washed, then incubated with 200 μM cholesterol for 1 h at 37 °C. The cells were then incubated with PRV at 100 TCID_50_ for 2 h at 4 °C.

For studies on cell entry, the cells were mock treated or pretreated with 8 mM MβCD at 37 °C for 30 min then infected with PRV(100 TCID_50_) for 1 h at 37 °C, after which remaining virus was removed by washing. The cells were kept in serum-free DMEM at 37 °C until mock treated, virus-infected cells showed complete CPE. For cholesterol replenishment, cells were pretreated with 15 mM MβCD at 37 °C for 30 min, washed and then incubated with exogenous cholesterol (200 μM) for 1 h at 37 °C.

For the post-entry study, cells were infected with PRV (100 TCID_50_) for 1 h at 37 °C, and the remaining virus was removed by washing. The cells were subsequently mock treated or pretreated with 8 mM MβCD at 37 °C for 30 min, washed in cold PBS and then incubated in DMEM at 37 °C until the mock treated, virus-infected cells showed complete CPE. For cholesterol replenishment, the cells were treated as described for the cell entry studies. Western blots and virus titers were performed to assess the effects of pre- and post-treatment on virus infection.

## Results

### Effects of cholesterol depletion on PRV infection

**Fig. 1:**
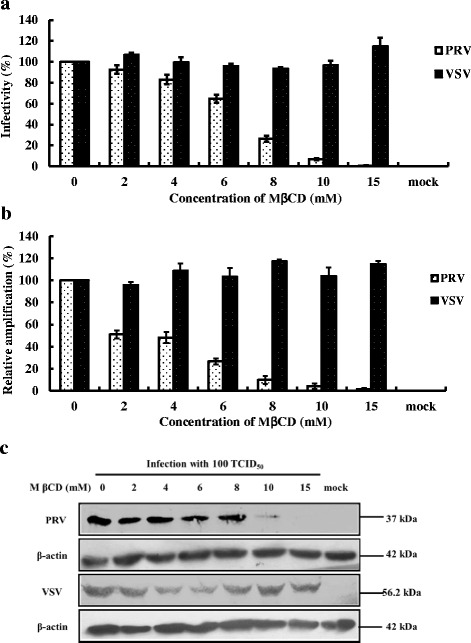
PRV infection inhibited by cholesterol depletion with MβCD. MA104 cell and BHK-21 cell monolayers were treated with increasing concentrations of MβCD before virus infection. Cells were subsequently, infected with PRV or VSV (100 TCID_50_) respectively, except “mock” as control without virus infection and MβCD treatment. **a** IFA data demonstrating infectivity of PRV and VSV using anti-VP7 of PRV antibodies and anti-G protein of VSV antibodies respectively. The 100% infectivity values of PRV and VSV represent average fluorescein-stained cells numbers of 100 and 240, respectively. **b** Real-time PCR data for PRV and VSV targeting the VP7 gene and G-protein. **c** Western blot analysis using antibodies to VP7 for PRV, G-protein for VSV and β-actin served as a protein loading control. Error bars indicate the standard deviations of three independent experiments

To determine whether the cholesterol component of lipid rafts plays a role in PRV infection, the cholesterol level in cellular membranes of MA104 cells for PRV and BHK-21 cells for VSV, respectively, were depleted by treatment with MβCD before infecting with PRV or VSV. After 24 h, VP7 polyclonal antibody for PRV binding exhibited a dose-dependent decrease in MA104 cells pretreated with increasing concentrations of MβCD (Fig. 1a). At a concentration of 15 mM, the infection rate of PRV decreased by 99%. In contrast, the infection rate of cholesterol-depleted BHK-21 cells infected with the control VSV was not affected. Real-time PCR results to quantify the PRV VP7 and VSV G gene, the viral load showed a similar response in MβCD-treated cells (Fig. 1b). Western blot (Fig. 1c) also demonstrated that depletion of cholesterol reduced the level of PRV infection in a dose-dependent manner where VP7 protein expression was nearly absent from MA104 cells at 15 mM MβCD. In contrast, no significant inhibitory effects were observed in MβCD treated BHK-21 cells infected with VSV as monitored by VSV G protein levels (Fig. 1c).

### Effects of cholesterol replenishment on PRV infection in cholesterol-deficient cells

**Fig. 2:**
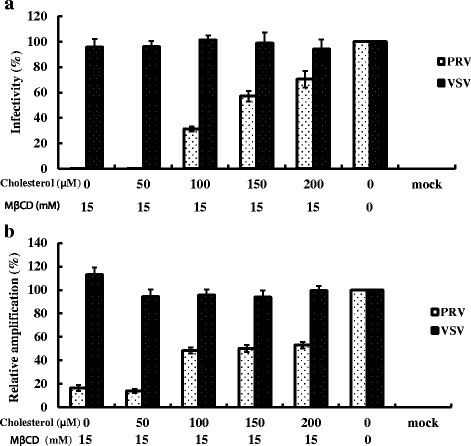
The restoration of PRV-infectivity by exogenous cholesterol. MA104 cell and BHK-21 cell monolayers were treated with 15 mM MβCD before supplemented with exogenous cholesterol. Subsequently, cells were infected with PRV or VSV (100 TCID_50_) respectively, except mock as control without virus infection and MβCD/ exogenous cholesterol treatment. **a** IFA data demonstrating infectivity of PRV and VSV using anti-VP7 antibodies and anti-VSV G protein antibodies respectively. The 100% infectivity values of PRV and VSV represent average fluorescein-stained cells numbers of 160 or 230, respectively; **b** Real-time PCR data for PRV and VSV targeting the VP7 and G protein gene. Error bars indicate the standard deviations of three independent experiments

The effects of PRV-infection on MA104 cells depleted of cholesterol with MβCD (15 mM) then replenished with exogenous cholesterol prior to PRV infection, are shown in Fig. 2. Virus levels were examined directly by IFA assay (Fig. 2a) and indirectly by RT-PCR (Fig. 2b). PRV infectivity as examined by IFA, increased in a dose-dependent manner and was restored to 70% of pretreatment levels (no MβCD) (Fig. 2a) with a concentration of 200 μM cholesterol. From the RT-PCR, we observed a similar, dose-dependent increase in the level of VP7 gene of PRV as the amount of exogenous cholesterol was increased (Fig. 2b), the VP7 gene expression reached 55% pretreatment levels (no MβCD).

### Co-localization of PRV VP7 protein and GPI-anchored protein

**Fig. 3:**
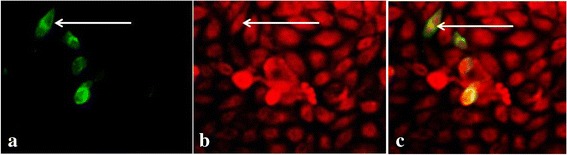
Co-localization of PRV VP7 protein and plasmid-derived, recombinant GPI-anchored protein. MA104 cells were treated for IFA as follows: **a** cells transfected with a plasmid expressing GPI-anchored green fluorescence labeling; **b** PRV infected cells incubated with anti-VP7 protein antibodies and counterstained with FITC second antibody; **c** co-localization of GPI-anchored protein and PRV VP7 protein on the cell membrane (Fig. 3a and b merged). Yellow color depicts overlapping binding

GPI-proteins have a predilection for lipid rafts. MA104 cells transfected first with GPI-pEGFP then with PRV (100 TCID_50_) showed a co-localization of both the recomninant GPI-protein (green) (Fig. 3a) and the PRV VP7 protein (red) (Fig. 3b) in dense regions of the cell membrane. These regions appeared yellow in merged figures (Fig. 3c). Data suggest that the PRV VP7 protein was localized in the lipid rafts of MA104 cells.

### Cholesterol is not required for PRV adsorption

**Fig. 4:**
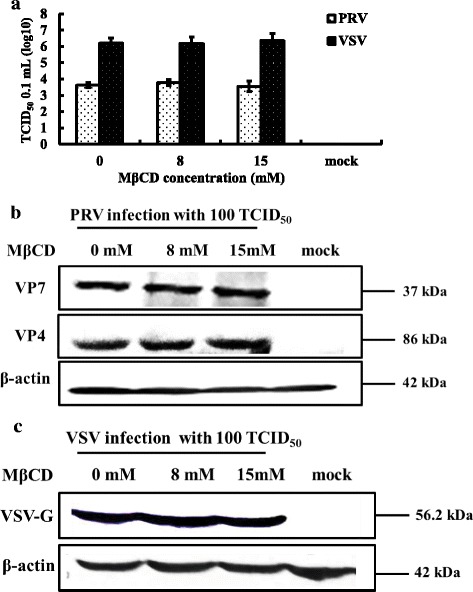
Cellular cholesterol and PRV adsorption. MA104 cells (PRV) and BHK-21 cells (VSV) were either mock treated or treated with 8 mM or 15 mM MβCD for 30 min, then infected with PRV or VSV (100 TCID_50_) at 4 °C for 2 h. Antibodies to the PRV VP7 or VP4 proteins and to the VSV G protein were used to screen western blots of cell homogenates and virus titers. **a** Virus titers of PRV and VSV after treatment. **b** Western blot of PRV VP7 and VP4 proteins and β-actin. **c** Western blot of VSV G protein and β-actin. Western blots depict representative experiments. Error bars (Fig. 4a) indicate the standard deviations of three independent experiments

To determine if cholesterol is important in PRV adsorption, cholesterol-depleted cells were incubated with PRV for 24 h at 4 °C to prevent viral replication, and then assayed for viral load by TCID_50_ and western blot. As shown in Fig. 4a and b, relative to mock-infected groups, no remarkable inhibitory effects were observed by TCID_50_ or western blot in the PRV-infected groups. The results mirrored those obtained with VSV (Fig. 4a and c) which is not affected by cholesterol levels. Data suggest that adsorption onto the cell membrane was not affected in cholesterol-depleted cells.

### Cholesterol depletion inhibits PRV entry in a reversible manner

**Fig. 5:**
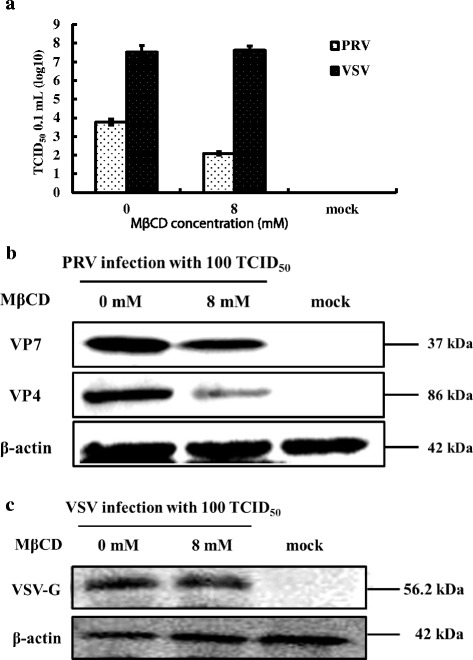
Cholesterol and control of PRV entry. MA104 cells (PRV) and BHK-21 cells (VSV) were either mock treated or treated with 8 mM MβCD for 30 min then infected with PRV or VSV (100 TCID_50_) for 1 h at 37 °C. Antibodies to the PRV VP7 or VP4 proteins and to the VSV G protein were used to screen western blots of cell homogenates and virus titers. **a** Virus titers of PRV and VSV after treatment. **b** Western blot of PRV VP7 and VP4 proteins and β-actin. **c** Western blot of VSV G protein and β-actin. Western blots depict representative experiments. Error bars (Fig. 5a) indicate the standard deviations of three independent experiments

To study the effects of cholesterol on PRV entry, cholesterol-depleted MA104 cells were incubated with PRV at 37 °C rather than 4 °C then viral loads examined 24 h post-infection. Results show that PRV virus titers (Fig. 5a) and viral protein levels (Fig. 5b) were decreased. At a concentration of 8 mM MβCD, we observed an approximate reduction in the virus titer of 50%, relative to controls. Expression levels of PRV viral protein were also significantly reduced; however, VSV and β-actin were not changed (Fig. 5c).

**Fig. 6:**
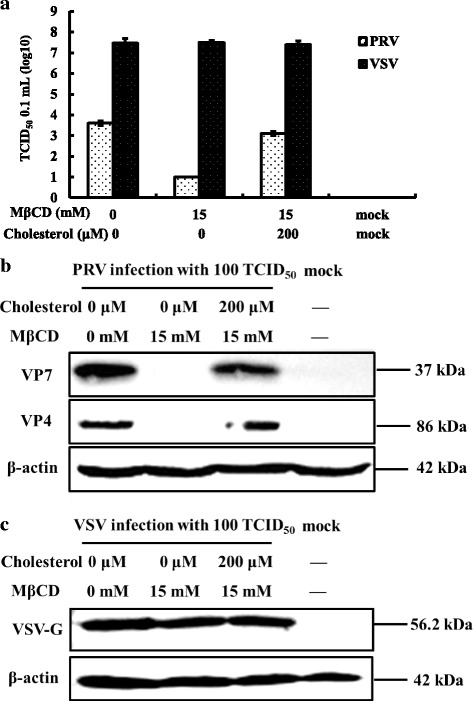
Reversing the effects of MβCD on PRV entry using exogenous cholesterol. MA104 cells (PRV) and BHK-21 cells (VSV) were pretreated with 15 mM MβCD at 37 °C for 30 min, then replenished 200 μM exogenous cholesterol at 37 °C for 1 h. Treated cells were then infected with PRV or VSV (100 TCID_50_), respectively, and the homogenates examined by western blot analysis and virus titers. **a** Virus titer of PRV and VSV after treatment. **b** Western blot of PRV VP7 and VP4 proteins and β-actin. **c** Western blot of VSV G protein and β-actin. Western blots depict representative experiments. Error bars (Fig. 6a) indicate the standard deviations of three independent experiments

The results on virus entry were reversible (Fig. 6). Cholesterol-depleted MA104 cells treated with 200 μM exogenous cholesterol for 1 h showed complete restoration of PRV virus titers relative to mock treated cells (Fig. 6a). The viral protein levels were increased significantly (Fig. 6b). Levels of VSV were not affected (Fig. 6a and c). Results suggest that stages following adsorption and most likely virus entry have a strict requirement for cholesterol.

### Cholesterol depletion of MA104 cells inhibited PRV post-entry stage

**Fig. 7:**
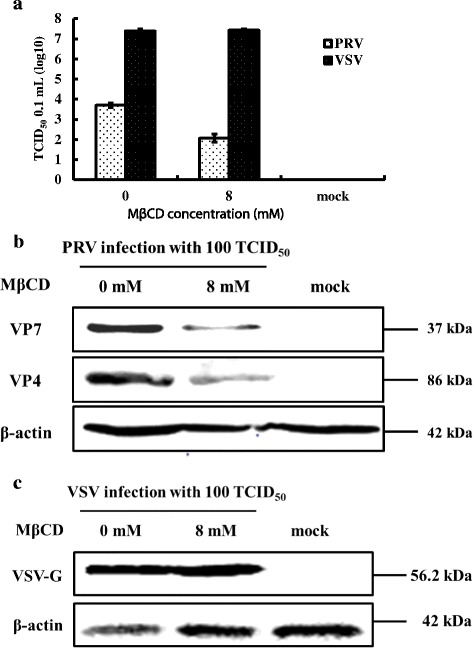
Cholesterol and control of PRV post-entry. MA104 cells (PRV) and BHK-21 cells (VSV) were first infected at 37 °C for 1 h then washed to remove extra virus. Cells were then treated with 8 mM MβCD incubated at 37 °C for 24 h. Antibodies to the PRV VP7 or VP4 proteins (PRV) and to the VSV G protein (VSV) were used to screen Western blots of cell homogenates. **a** Virus titer of PRV and VSV after treatment. **b** Western blot of PRV VP7 and VP4 proteins and β-actin. **c** Western blot of VSV G protein and β-actin. Western blots depict representative experiments. Error bars (Fig. 7a) indicate the standard deviations of three independent experiments

**Fig. 8:**
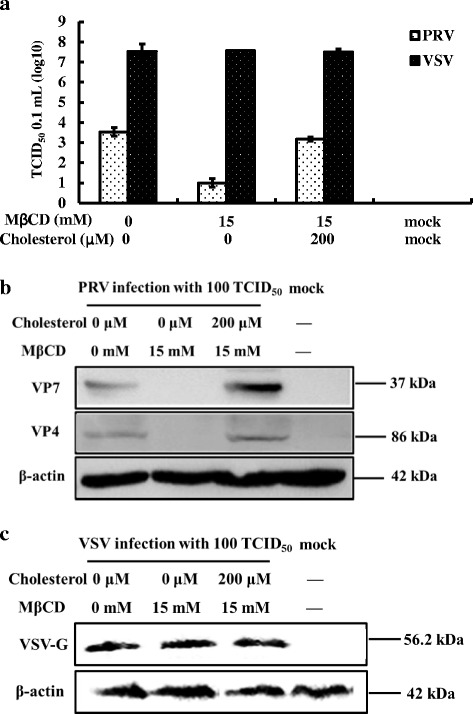
Reversing the effects of MβCD on PRV post-entry using exogenous cholesterol. MA104 cells (PRV) and BHK-21 cells (VSV) that had been infected and treated with MβCD were then incubated in the presence of 200 μM exogenous cholesterol. Western blots and virus titers were performed to assess the effects of pre- and post-treatment on virus infection. **a** Virus titer of PRV and VSV after treatment. **b** Western blot of PRV VP7 and VP4 proteins and β-actin. **c** Western blot of VSV G protein and β-actin. Western blots depict representative experiments. Error bars (Fig. 8a) indicate the standard deviations of three independent experiments

To analyze post-entry stages, MA104 cells were first infected with PRV then treated with MβCD. As shown in Fig. 7a, b, and c, the PRV virus titers and viral protein levels decreased relative to VSV controls, whereas replenishing exogenous cholesterol restored virus titers to pretreatment levels relative to VSV controls (Fig. 8a). The viral proteins were also increased significantly (Fig. 8b) relative to VSV controls (Fig. 8c) demonstrating that post-entry stages were also controlled by the level of cellular cholesterol.

## Discussion

Lipid rafts are densely packed, floating asemblages of cholesterol, sphingolipid and mostly receptor proteins that are present within cell membranes and that function in membrane trafficking and signaling [30]. Among other things, lipid rafts play critical roles in viral entry, replication, assembly and budding, as well as in protein transport [31]. Cholesterol is a key component of lipid rafts that compartmentalize cellular processes. Further, changes in cellular cholesterol levels have been linked to alterations in the infection process of numerous viruses where the cholesterol dissipative agent, MβCD, has been used to evaluate cholesterol-enriched lipid rafts in virus infection. MβCD is a strictly surface-acting agent that rapidly removes cholesterol from the plasma membrane. Some non-enveloped viruses, such as members of *Picornaviridae* and *Reoviridae,* are assembled in the cytoplasm and released by cell lysis. However, evidence has been advanced showing that exit from infected cells can occur in the absence of cell lysis, suggesting that alternate paths may be utilized for egress by non-enveloped viruses [4]. Other members of the non-enveloped virus, such as RRV and bluetongue virus (BTV), along with a number of enveloped viruses such as transmissible gastroenteritis virus (TGEV) [27], porcine reproductive and respiratory syndrome virus (PRRSV) [32], porcine pseudorabies virus (PrV) [33], Rift valley fever virus [34] have been shown to be sensitive to MβCD treatment of host cells. Further, RRV and BTV have been shown to interact with lipids during the progress of infection suggesting the importance of cholesterol-rich microdomains in the infection process. The entry of rotaviruses (RV) into host cells is a multistep process. Different rotavirus strains display different requirements for host cells. Some strains depend on the presence of sialic acid (SA) on the cell surface [35]. Others have demonstrated a requirement for several integrin during infection. As example, the VP4 protein of RV contains tripeptide sequence motifs for integrin α2β1 and α4β1, whereas VP7 contains ligand sites for integrin αxβ2 and α4β1 [36, 37]. In addition, heat shock protein and certain gangliosides were identified as cellular molecules associated with RV entry.

The non-enveloped PRV, is a leading etiologic cause of severe dehydrating diarrhea in piglet worldwide. Consequently, there is an urgent need to develop effective preventive and therapeutic strategies to combat this pathogen. Currently, it is unknown whether or not cholesterol-enriched lipid rafts which is present in the membranes of the host cells, is required for PRV infection, and if so, how it is associated with PRV infection. To address this question, cholesterol in the cellular membrane of MA104 cells was removed by MβCD treatment prior to PRV infection. Results demonstrated that lipid rafts depleted of cholesterol decreased the infectivity of PRV in a dose-dependent manner. Conversely, replenishment of cholesterol partially restored viral infection. These results are similar to those observed on RRV [14], BTV and poliovirus [6] which are also non-enveloped viruses [5, 38].

Our results collectively demonstrate that PRV infection interacts with cholesterol-enriched lipid rafts. First, direct treatment of the virus with MβCD prior to infection had no effect in the infection process (data not shown) indicating the observed effects were unrelated to interactions between the drug and the virus. Second, the concentration of MβCD and cholesterol used in this study did not generate significant adverse effects on cell viability as shown by MTT (data not shown). Third, the drug concentrations and the protocols are similar to those described in studies involving other viruses [27, 39]. Finally, co-localization studies showed that PRV VP7 protein and a recombinant GPI-anchor protein which has a predilection for lipid rafts, were localized to the lipid rafts in the plasma membranes of MA104 cells.

Studies on the key stages of the PRV infection process requiring cholesterol have not been reported. In this study, we showed that adsorption of the virus onto the cell membrane at low temperatures was not affected by depletion of cholesterol; no significant differences were observed between MβCD-treated cells and mock cells. However, when cells were treated with MβCD prior to or after virus entry, PRV infection declined in a dose-dependent manner. The replenishing of exogenous cholesterol reversed these affects indicating that cholesterol depletion affected mechanisms involved in the entry of the virus and one or more mechanism downstream of virus entry. Similar results were also observed with the bovine rotavirus (BRV) where cholesterol depletion significantly impaired BRV entry and assembly but did not reduce BRV replication [39]. These results suggested that PRV entry may be inhibited by cholesterol depletion though disturbing lipid-raft-dependent endocytosis.

In summary, lipid rafts play vital roles in PRV infection, and plasma membrane-based cholesterol is a key determinant for entry and post-entry stages of PRV infection. By depleting plasma membrane cholesterol we were able to provide a direct link between cholesterol levels, lipid rafts and the ability of PRV to infect host cells. Future studies are needed to investigate synergistic effects between MβCD and other drugs capable of disrupting lipid rafts in porcine intestinal epithelial cell cultures.

## Conclusions

Our results reveal the mechanism of PRV infection that membrane-cholesterol localized to lipid rafts is an indispensable biomolecule for PRV infection during the entry and post-entry into the cells rather than virus binding on cell surface; on the other hand, MβCD, a cellular cholesterol-depletion agent, inhibit the PRV infection on the susceptible cells, have a huge potential to develop a new anti-viral strategy.

## Abbreviations

BRV: Bovine rotavirus
BTV: Bluetongue virus
CPE: Cytopathic effects
DMEM: Dulbecco’s Modified Eagle Medium
FCS: Fetal calf serum
GPI: Glycosyl-phosphatidylinositol
h: Hours
HIV: Human immunodeficiency virus
HRP: Horseradish peroxidase
IFA: Indirect immunofluorescence assay
min: Minute
MTT: Mosmann based assay
MβCD: Methyl-β-cyclodextrin
PBS: Phosphate-buffered saline
PRRSV: Porcine reproductive and respiratory syndrome virus
PrV: Porcine pseudorabies virus
PRV: Porcine rotavirus
RRV: Rhesus rotavirus
SA: Sialic acid
SV40: Simian virus 40
TBST: Tris-buffered saline containing 0.05% Tween 20
TCID_50_: The 50% tissue culture infectious dose
TGEV: Transmissible gastroenteritis virus
VSV: Vesicular stomatitis virus
